# The role and utility of artificial intelligence and machine learning for diagnostic prediction in general practice

**DOI:** 10.1080/13814788.2026.2620908

**Published:** 2026-02-02

**Authors:** Liesbeth Hunik, Annemarie A. Uijen, Jacqueline K. Kueper, Amanda L. Terry, Tim C. olde Hartman, Twan van Laarhoven, Henk J. Schers

**Affiliations:** ^a^ Department of Primary and Community care, Research Institute for Medical Innovation, Radboudumc, Nijmegen, The Netherlands; ^b^ Scripps Research Translational Institute, Scripps Research, San Diego, CA, USA; ^c^ Centre for Studies in Family Medicine, Department of Family Medicine, Department of Epidemiology & Biostatistics, Schulich School of Medicine & Dentistry, Western University, London, ON, Canada; ^d^ Institute for Computing and Information Science, Radboud University, Nijmegen, The Netherlands

**Keywords:** Artificial intelligence, machine learning, diagnostic prediction, traditional statistics, primary care, general practice, decision support

## Abstract

Diagnostic prediction models are commonly used in general practice to support clinical decision-making. Traditionally, these models have been developed using statistical methods such as logistic regression. While these approaches have proven useful, they often produce average risk estimates that may not fully account for the complexity of individual patients. In recent years, the use of machine learning (ML), a subfield of artificial intelligence (AI), has grown in healthcare. We examine the similarities and differences between traditional statistical methods and AI/ML approaches for diagnostic prediction in general practice. Using examples from daily practice, we explore how ML techniques can add value, particularly in handling large, complex datasets such as those derived from electronic health records. We also discuss key challenges that hinder the adoption of AI/ML in general practice, including interpretability, data quality, external validation, clinical relevance, implementation and legal issues, and practical usability. We provide recommendations to overcome these challenges. The potential of AI/ML can only be realised if tools are developed collaboratively with GPs, focused on real-world clinical problems, and rigorously validated in practice settings. GP associations, GPs, patients, and primary care scientists should take an active role in the development, validation, and implementation of AI/ML-based diagnostic prediction tools for general practice.

## Introduction

In recent years, artificial intelligence (AI) and machine learning (ML) have attracted growing attention in healthcare [1]. AI has no official definition but is often described as the ability to simulate human intelligence [2]. ML, a subfield of AI, includes a variety of different statistical and mathematical techniques to learn patterns from data [2]. Although the concepts of ML and AI are often used interchangeably, ML should be regarded as a method to achieve artificial intelligence. The application of AI/ML to diagnostic prediction in primary care is expanding, offering new possibilities to support general practitioners (GPs) in their clinical decision-making and personalised care. A recent paper in this journal has emphasised the potential of generative AI for European primary care research, particularly its role in supporting clinical decision making [3].

Diagnostic prediction is an important part of clinical decision making. It focuses on determining the patient’s diagnosis rather than addressing therapeutic interventions or predicting prognosis. During clinical encounters in everyday practice, GPs often consider the probability of diagnoses for each patient based on both implicit and explicit epidemiological knowledge. For some diagnoses, GPs can be supported by using prediction models, such as the Wells’ criteria for pulmonary embolism [4], or the gout calculator for gout [5]. These models predict the probability of a disease based on multiple variables or predictors [6]. Traditional statistical methods are widely used to develop these models. AI is suggested as a promising partner in these clinical decision-making processes [7]. AI/ML-based prediction models are potentially able to include more individual patient variables (e.g. patient’s medical history and additional laboratory results) and capture more complex patterns to better tailor risk estimates to the unique circumstances of each patient. Previous diagnostic prediction tools have been developed through AI/ML use that can predict the risk of diseases, such as dementia [8] and colorectal cancer [9]. These models are able to predict dementia and colorectal cancer based on patient data.

In this paper, we describe both the opportunities and challenges that AI/ML introduces. We believe that AI/ML has real potential, but successful implementation depends on aligning the development of tools with the needs and realities of GPs.

Box 1. Opportunities and challenges of AI/ML in diagnostic prediction

Opportunities

- *Early diagnosis;* diagnosis of conditions in earlier stages of a disease
- *Diagnostic process*; assistance in pattern recognition and differential diagnosis
- *Risk assessment*; early recognition of risk factors for chronic and serious diseases in EHR data

Challenges

- *Interpretability and explainability; knowing* how to use the tool and interpret the results
- *Quality and amount of data;* proper development and evaluation of AI/ML tools is necessary to prevent biased outcomes
- *External validation;* testing AI/ML tools on real-world data
- *Legal concerns*; AI/ML in clinical practice comes with safety and legal challenges
- *Relevance and practicality;* potential applications are numerous, focus on prediction tools relevant and practical for general practice

## Key principles of using AI/ML for diagnostic prediction in general practice

There are many different AI techniques, ranging from traditional ML and deep learning to large language models and generative AI. ML includes numerous different statistical and mathematical modelling techniques [2]. The distinction between traditional statistics and ML is not always clear. Some ML techniques resemble statistical methods, while others differ significantly. For example, logistic regression is considered to be statistics in some cases and is considered to be ML in others. In contrast, neural networks are considered very powerful ML and would not be typically classified as statistical techniques.

Whether a statistical or an AI method is appropriate for prediction research purposes, depends on the task or outcome that has to be predicted and the data which are available for input. In general practice, vast amounts of data can be drawn from electronic health records (EHR). EHR data contain a variety of health information about a patient’s life: medical history, investigations, prescriptions, interventions, and contextual knowledge. EHR data is aggregated and contains information in different formats (text data, illnesses in a disease code like the International Classification of Primary care (ICPC) and blood pressure in numbers). This makes EHR data messy and unstructured. AI/ML techniques are ideal for making these complex data usable and valuable supporting GPs in daily practice [10]. For example, consider textual medical history data. While this unstructured data cannot be used as input for statistical methods, it can be used as input for several AI methods (e.g. large language models). Therefore, AI-based diagnostic prediction models may be able to predict a more precise outcome for patients with extensive medical backgrounds by using more variables from history-taking, physical examinations and many other parameters in the patient’s file [11].

The potential of AI/ML techniques lies therefore mainly in their ability to make more precise predictions at an individual level, recognise complex patterns, and handle complex data sources, such as large amounts of EHR data. There are a lot of different AI techniques from traditional ML to generative AI and the choice of technique depends on the data input and the intended use of the model or objective of an analysis.

## The current state of AI/ML diagnostic prediction tools in general practice

Although the use of AI/ML-based diagnostic prediction tools in general practice is still in its early stages, several practical applications have already found their way to daily practice. Currently, most tools in use serve to assist clinicians in pattern recognition, risk assessment, and triage rather than providing definitive diagnoses [12]. Examples of tools used in clinical settings include AI algorithms that analyse electrocardiograms (ECGs) to detect atrial fibrillation or other arrhythmias [13], and tools that interpret skin lesion images to support early detection of melanoma [14]. In laboratory medicine, AI models are also increasingly used to interpret blood test patterns that may indicate specific conditions such as kidney injury [15]. However, in most cases, AI/ML applications are still used in pilot settings or within research projects rather than routine practice [16–19]. Most models have not been proven suitable for implementation in daily general practice yet [18].

## Opportunities for AI/ML in diagnostic prediction

Although the potential of integrating AI/ML into general practice is promising, GPs express mixed feelings about the potential [1,20]. Many see the potential of using ML in daily practice, but are still hesitant because they want to know if prediction models perform sufficiently and want to understand the results of a ML model well enough before incorporating AI based prediction models into daily practice [1]. The opportunities for AI/ML lie in improving diagnostic accuracy, in order to improve clinical decision making and support personalised care. To illustrate the potential for primary care, examples of approved diagnostic prediction models that are classified as medical devices are mentioned. Diagnostic accuracy is supported through:

1. Early diagnosis. AI/ML diagnostic prediction tools for medical problems can assist the GP in complex cases that are hard to diagnose [21]. Prediction tools also have the potential to diagnose conditions before symptoms become more severe. Prediction models have been developed that provide early detection of diseases based on EHR data, such as for lung cancer [22] or COVID-19 [23].
2. Correct diagnosis or reducing misdiagnosis. AI/ML diagnostic prediction tools can support GPs in predicting the correct diagnosis. A symptom checker, for example, can predict likely diseases based on patient’s symptoms and medical history. Such tools may guide GPs towards the correct diagnosis and enable earlier detection. Examples of symptom checkers using AI/ML algorithms are ADA health [24] and Symptomate [25].
3. Early risk recognition. Diagnostic prediction tools are able to assist in detecting drug-related problems with a tool that automatically signals problems for patients with chronic conditions or those using certain types of medication [21,26,27]. For example, when a patient uses medication for diabetes and is vomiting, this causes an increased risk of dehydration. The combination of certain drugs and dehydration is a risk and may be easily overlooked. A diagnostic prediction tool could recognise these risks and provide a real-time alert in the EHR to notify the GP.
4. Pattern recognition for imaging or lab results. This relates to both reducing misdiagnosis and early risk recognition. Prediction tools can find patterns for certain diseases in lab results or the EHR and can help match the signs and symptoms of patients with these disease specific patterns. One example in a hospital setting is the Sepsis ImmunoScore (US) for prediction of sepsis after hospital admission [28].

## Challenges for AI/ML in diagnostic prediction

Most AI models that have been developed have not been implemented in general practice. This means we still face multiple important challenges before large-spread implementation of AI in general practice will be possible. These can be divided in six main groups:

1. Interpretability and explainability. Interpretability refers to understanding how the model processes input. It can be hard to understand the patterns the AI/ML technique learned to make the most accurate prediction. Explainability refers to being able to clarify or justify the results of the model [29]. The Wells criteria assign points based on specific clinical features and risk factors; the total score determines the probability category. Understanding which points correspond to which clinical features can be considered interpretability, while communicating to a patient the implications can be considered explainability. Some ML techniques lack interpretability and explainability more than others. It is important that the provider knows how to use the diagnostic support tool and how to interpret the results [11,30]. A few papers have been published that explain how to understand and interpret ML [10,11,30,31]. A lack of comprehension of the results causes users of the tool to rely too much on the prediction of the tool [11].
2. Quality and amount of data. The performance of an AI/ML tool is as good as the data it uses [26,27,32]. This is, for example, important in predicting rare diseases, because a tool will not be able to learn the patterns necessary to make good predictions if the dataset is too small. Similarly, if a particular type of patient population is not well represented in the training data or if the training data contain biases themselves the AI/ML model may make biased predictions. For example, insufficient data is often seen for ethnic differences. This can result in underestimation or overestimation of a particular condition in a particular ethnic group. Proper development and evaluation of ML models is necessary to avoid exacerbation of underrepresented groups in the data [10].
3. External validation. Most ML tools perform well in retrospective studies but are tested only on the dataset that they were developed from [16]. They may perform less well when applied to new data or real clinical settings. Before a tool can be implemented in daily practice, it should be tested with an external dataset (external validation). With external validation you can detect overfitting. Overfitting occurs when a model learns the relevant and irrelevant patterns in the training data [30]. External validation often results in low performance or is not even conducted [16,18,30]. This results in diagnostic prediction tools that are less usable. The two models from the introduction that were able to predict dementia [8] and colorectal cancer [9] based on EHR data, were not externally validated. Therefore, it is unknown how these models perform on real-world data.
4. Implementation and legality. Following external validation, implementation in real-world clinical practice introduces further safety and legal challenges. These include determining the acceptable level of accuracy and clarifying responsibility if the tool produces an incorrect recommendation [33]. This is regulated in the EU medical device regulation (MDR) [34]. However, obtaining EU MDR approval involves numerous steps and considerable time, presenting significant challenges for researchers or companies developing medical devices [35]. This may lead to GPs using medical devices without EU MDR certification, which could pose risks for patients if the results are not interpreted with appropriate caution.
5. Relevance. The frequent challenge is the current misalignment between the AI application and the requirements for general practice [33]. Research has shown that GPs are rarely involved in the process of developing a diagnostic prediction tool and that tools are often only tested in research/technical setting and not in a general practice [16,18,19]. This can result in a tool for a non-relevant problem, for example a tool to predict a disease that is easy to diagnose based on a simple blood test. The patient is often even less involved in the process than the GP [16]. It is a responsibility for developers that GPs need to be able to rely on prediction tools that have been appropriately and thoroughly evaluated [21,33]. GPs also share responsibility for ensuring relevant diagnostic prediction tools, which requires pro-active involvement in the development of tools [3]. There are currently a limited number of usable AI/ML based diagnostic prediction tools developed for GPs [18].
6. Practicality. As the number of AI tools increases, their practical integration becomes a major challenge. Many prediction tools are developed for a single disease or outcome, limiting their flexibility [16]. This makes them less practical, since GPs would need multiple tools for different diseases. Prediction tools should ideally integrate into electronic health records, providing real-time risk scores or alerts to be truly useful for GPs [3].

## Recommendations for research and practice

To move beyond the challenges, it is important to be aware of the quality and clinical relevance of data in the early stage of the development of a diagnostic prediction tool. Researchers should carefully examine their datasets for potential biases that could influence the results of their research question. We recommend using independent datasets for the validation of diagnostic prediction model. Successful implementation depends on aligning the development of tools with the needs and practical realities of GPs. New tools should be developed in close collaboration with GPs, focus on real-world clinical problems, and be rigorously validated in general practice settings. GPs should take a leading role in every step of the development, validation, and implementation of diagnostic prediction models [3]. More research is needed, and frameworks like TRIPOD-AI can be used to report findings of the developed prediction tool [36]. The opportunities and challenges provide a foundation for collaborative teams of GPs, IT professionals, and data scientists, whose involvement is essential at every stage of developing, validating, and implementing an AI/ML-based diagnostic prediction model [3,7,33].

## Conclusion

AI/ML techniques offer promising opportunities to support diagnostic prediction in general practice by enabling more individualised, data-driven decision support. However, several challenges must be addressed before AI-supported diagnostic prediction can be widely used in routine care. These challenges led to our recommendations for research and practice. Tools should focus on clinically relevant problems and should be externally validated in real-world settings. Only then can AI/ML meaningfully contribute to the diagnostic process in general practice. Since diagnostic prediction tools are mostly not EU MDR certified, outcomes of such tools should be handled cautiously. Further research is needed, and GP associations, such as the Dutch College of GPs, GPs themselves, patients, and primary care scientists should take an active role in the development, validation, and implementation of AI/ML-based diagnostic prediction tools for general practice.
